# Fatal Neutropenic Enterocolitis in a Young Female After the First Round of Eculizumab for Paroxysmal Nocturnal Hemoglobinuria

**DOI:** 10.7759/cureus.69088

**Published:** 2024-09-10

**Authors:** Laura Miranda-Burgos, Aliya Khan, Gautam Anand, Nemer Dabage-Forzoli

**Affiliations:** 1 Internal Medicine, Broward Health North, Deerfield Beach, USA; 2 Gastroenterology, Broward Health North, Deerfield Beach, USA; 3 Internal Medicine, University of Miami at Holy Cross, Fort Lauderdale, USA

**Keywords:** aplastic anemia, eculizumab, immunodeficiency, multiorgan failure, neutropenic enterocolitis, paroxysmal nocturnal hemoglobinuria, sepsis

## Abstract

Paroxysmal nocturnal hemoglobinuria (PNH) is a rare disorder characterized by glycosylphosphatidylinositol (GPI)-linked membrane protein deficiency, leading to hemolytic anemia and thrombosis. A subset of patients develop severe neutropenia, predisposing them to neutropenic enterocolitis (NEC). We present a case of fatal NEC following eculizumab therapy in a 31-year-old female with PNH. She presented with abdominal pain, fever, and neutropenia post-eculizumab. Despite aggressive management, including antibiotics and supportive care, she developed septic shock complicated by bacteremia, multiorgan failure, and two episodes of cardiac arrest, leading to severe lactic acidosis and ultimately progressing to brain death. The etiology of NEC in PNH patients remains multifactorial, involving immunocompromised and treatment-related factors. This case underscores the challenges in managing NEC and highlights the importance of early recognition and intervention in high-risk patients.

## Introduction

Paroxysmal nocturnal hemoglobinuria (PNH) is a rare hematologic disorder, with an incidence of approximately 1-1.5 cases per million individuals globally. It is characterized by an acquired deficiency of glycosylphosphatidylinositol (GPI)-anchored proteins on the cell membrane, resulting in intravascular hemolysis, thrombosis, and peripheral blood cytopenias [1]. While the clinical spectrum of PNH primarily encompasses hemolytic anemia and thrombotic events, a subset of patients, less than 4%, may experience severe neutropenia, predisposing them to potentially life-threatening complications such as neutropenic enterocolitis (NEC) [2-8]. NEC, characterized by necrotizing inflammation of the bowel wall, predominantly affects the cecum, terminal ileum, and ascending colon, posing a significant clinical challenge, particularly in immunocompromised individuals [3].

Eculizumab, a monoclonal antibody targeting complement protein C5, has emerged as a paradigm-shifting therapeutic agent in the management of PNH, significantly reducing hemolysis and thrombotic complications. However, its immunomodulatory effects and potential impact on gastrointestinal health have raised concerns regarding its role in precipitating gastrointestinal complications, including NEC [6]. Understanding the etiology of NEC in the context of PNH and eculizumab therapy necessitates a comprehensive evaluation of underlying disease pathology, therapeutic interventions, and host factors predisposing individuals to gastrointestinal complications. 

We describe a compelling case of fatal NEC following the initiation of eculizumab therapy in a young female with PNH. This case underscores the intricate interplay between underlying hematologic disorders, therapeutic interventions, and predisposing factors in the development of NEC. Through a detailed analysis of the clinical presentation, diagnostic findings, management strategies, and underlying pathophysiological mechanisms, we aim to elucidate the complexities surrounding the etiology and management of NEC in PNH patients receiving eculizumab therapy. This case serves as a poignant reminder of the challenges inherent in the management of gastrointestinal complications in immunocompromised patients with hematologic disorders and highlights the importance of a multidisciplinary approach to optimize patient outcomes.

## Case presentation

A 31-year-old Haitian female with a history of aplastic anemia secondary to PNH presents to the emergency department with a three-day history of severe diffuse abdominal pain, nausea, vomiting, diarrhea, fever, and chills, two days after receiving her first round of eculizumab for her underlying condition. Initial vital signs were significant for a temperature of 39.9°C, a heart rate of 145 beats per minute (bpm), a blood pressure of 144/72 mmHg, and a respiratory rate of 24 respirations per minute (rpm). The patient was saturating 94% on 2 liters of oxygen via nasal cannula. Physical examination was remarkable for an acutely ill-appearing female, diaphoretic, in severe distress due to pain, dry mucous membranes, and diffuse abdominal tenderness. Initial laboratory analysis (Table 1) was notable for a white blood cell count of 0.5 x 103/µL, an absolute neutrophil count (ANC) of 0 cells/µL, a hemoglobin of 7.1 g/dL, and a platelet count of 3 x 103/µL, meeting criteria for neutropenic sepsis. Blood chemistry revealed severe electrolyte derangements, transaminitis with hepatocellular injury pattern, and high anion gap metabolic acidosis.

**Table 1:** Laboratory analysis of the patient in reference to normal range. ANC: absolute neutrophil count

Parameter	Result	Reference range
White blood cells	0.49 x 10^3^/µL	4.00-11.00
ANC	0 cells/µL	2,500-6,000
Hemoglobin	7.1 g/dL	11.5-15.3
Platelets	3.0 x 10^3^/µL	140-400
Sodium	130 mmol/L	136-145
Potassium	2.8 mmol/L	3.5-5.1
Serum bicarbonate	8 mmol/L	22-29
Anion gap	18	5-14
Lactic acid	13 mmol/L	0.5-2.2
Alkaline phosphatase	64 unit/L	40-150
Aspartate aminotransferase	341 unit/L	5-34
Alanine aminotransferase	300 unit/L	0-55
Total bilirubin	0.9 mg/dL	0.2-1.2

**Figure 1:**
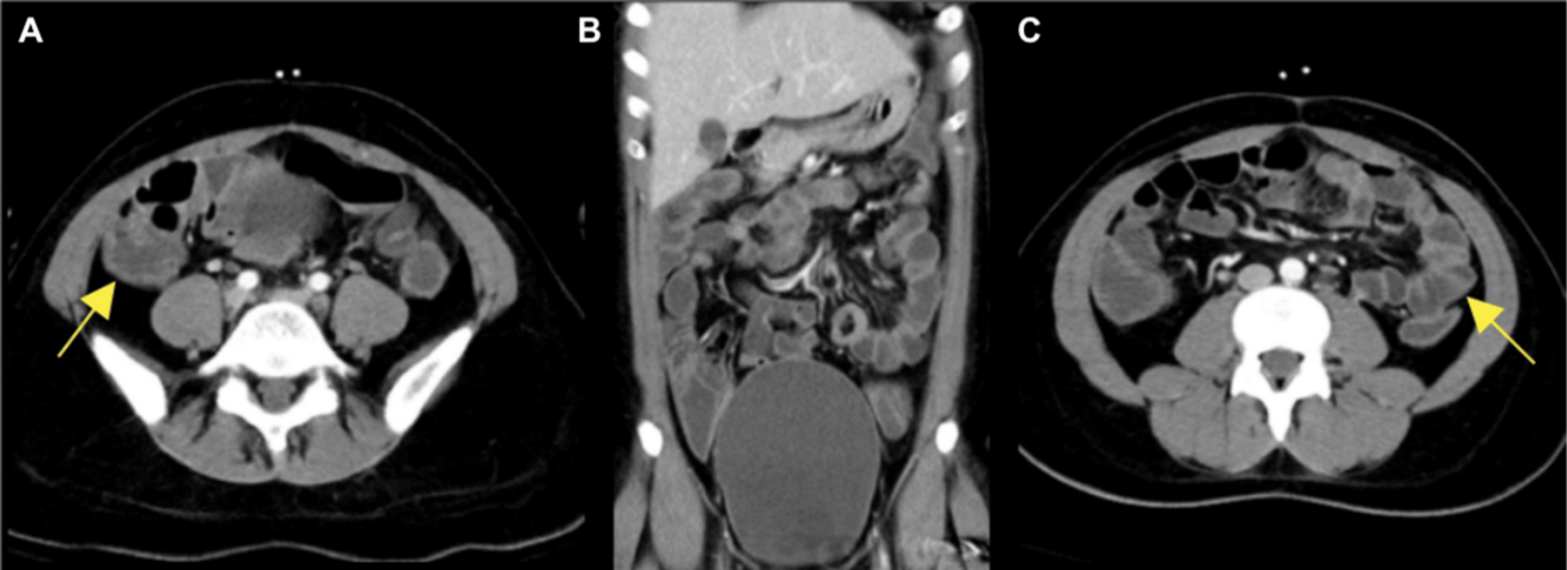
Axial (A, C) and coronal (B) CT images of the abdomen and pelvis with intravenous contrast demonstrating diffuse bowel wall thickening (yellow arrows) suggestive of enterocolitis.

A computed tomography (CT) scan of the abdomen and pelvis with intravenous contrast demonstrated diffuse bowel wall thickening, suggestive of enterocolitis (Figure 1). Thrombosis was excluded as a potential diagnosis.

Fluid resuscitation was promptly commenced. Blood cultures were collected before the initiation of empiric antibiotic therapy with intravenous vancomycin, cefepime, and metronidazole, in addition to micafungin for antifungal coverage. The patient was urgently transferred to the intensive care unit, where she received blood products and antipyretics. Filgrastim was administered for severe neutropenia. General surgery was consulted for signs of acute abdomen, including worsening abdominal distention, tenderness, and absent bowel sounds. Nasogastric tube decompression was attempted. Vasopressor support was initiated due to hemodynamic instability, and the patient was subsequently intubated due to acute hypoxic respiratory failure. Repeat blood work was notable for worsening pancytopenia. Surgical intervention was deferred at this time. 

The patient had a cardiac arrest likely secondary to metabolic causes and a return of spontaneous circulation was achieved. Blood cultures identified multidrug-resistant Klebsiella pneumoniae and Enterococcus cloacae. Intravenous antibiotic therapy was subsequently adjusted to meropenem and vancomycin for appropriate coverage. She developed multiorgan failure with worsening lactic acidosis and acute versus chronic disseminated intravascular coagulation. Continuous renal replacement therapy was initiated along with immediate transfusion of fresh frozen plasma, platelets, packed red blood cells, and cryoprecipitate. The patient had an asystole cardiac arrest, and despite being successfully resuscitated twice, she continued to deteriorate. A neurological exam revealed loss of brainstem reflexes. Magnetic resonance imaging (MRI) of the brain revealed anoxic brain injury, severe edema, and herniation, and she eventually succumbed to brain death.

## Discussion

Neutropenic enterocolitis (NEC), although rare, presents a considerable challenge in managing patients with underlying hematologic disorders such as paroxysmal nocturnal hemoglobinuria (PNH). In this case, the etiology of NEC prompts a nuanced inquiry into the interplay between the patient's underlying aplastic anemia, the immunocompromised state inherent to PNH, and the potential contributory role of eculizumab therapy. 

Aplastic anemia, a hallmark of PNH, is characterized by bone marrow failure and profound cytopenias, including neutropenia. The resultant immunocompromised state predisposes patients to infectious complications, including NEC [2]. The pathophysiology of NEC in this context likely involves mucosal injury and bacterial translocation due to the compromised gut barrier function, exacerbated by the underlying hematologic disorder. 

Eculizumab, a terminal complement inhibitor, has revolutionized the management of PNH by mitigating intravascular hemolysis and thrombotic events. However, its impact on the immune system remains a subject of debate. Eculizumab-mediated complement blockade may theoretically increase susceptibility to infections, including NEC, by impairing the opsonization and clearance of microorganisms [6]. Moreover, the infusion of monoclonal antibodies itself can trigger immune reactions and cytokine release, potentially exacerbating gastrointestinal inflammation [3,4].

This case substantiates the multifactorial nature of NEC, driven by the complex interplay of aplastic anemia, PNH-related immunocompromise, and eculizumab therapy. While the immunosuppressive milieu of aplastic anemia sets the stage for gastrointestinal complications, including NEC, the introduction of eculizumab may have further perturbed the delicate balance of immune homeostasis, tipping the scales towards gastrointestinal inflammation and bacterial translocation.

## Conclusions

In conclusion, the etiology of NEC in patients with PNH is likely multifactorial, involving the underlying hematologic disorder, the immunocompromised state, and the potential immunomodulatory effects of eculizumab therapy. The prognosis in this setting is typically poor if left untreated; however, with appropriate management and sufficient improvement in neutrophil count, patients may survive and achieve a favorable outcome. Clinicians should remain vigilant for gastrointestinal complications and implement a multidisciplinary approach. This should involve hematology for the management of PNH and eculizumab therapy, infectious disease for managing potential infections, nutrition for addressing dietary requirements, and surgical consultation when necessary. Such a comprehensive strategy is essential for optimizing management and improving patient outcomes.
